# Dynamic Biomarker Assessment: A Diagnostic Paradigm to Match the AKI Syndrome

**DOI:** 10.3389/fped.2019.00535

**Published:** 2020-01-21

**Authors:** Rajit K. Basu

**Affiliations:** Division of Critical Care, Department of Pediatrics, Emory School of Medicine, Children's Healthcare of Atlanta, Atlanta, GA, United States

**Keywords:** AKI, biomarkers, pediatrics, critical care, syndrome, NGAL, TIMP2/IGFBP7, FST

## Abstract

Acute kidney injury (AKI) affects one in four neonates, children, and adults admitted to the intensive care unit (ICU). AKI-associated outcomes, including mortality, are significantly worsened. Several decades of research demonstrate evidence for a need to rethink the pathophysiology and drivers of injury as well as to reconsider the existing diagnostic framework. Novel urinary and serum biomarkers of injury have, however, not been readily integrated into practice—partially because of the limited scope to current testing. The predominant focus to date has been the adjudication of a single biomarker measured at a single point of time for the prediction of either AKI progression or disease-related mortality. This approach is pragmatically problematic. The imprecise, umbrella classification of AKI diagnosis coupled with the absence of a consistently effective set of therapies creates a difficult rubric for biomarkers to demonstrate value in the scope of practice. AKI is, however, not a binary process but more an ICU syndrome—with complex biology underpinning injury, interacting and disrupting other organ function, multidimensional in manifestation, and varying in severity over time. As such, a more appropriate diagnostic paradigm is needed. In this minireview, the status quo for AKI diagnosis and associated limitations will be discussed, and a novel, dynamic, and multidimensional paradigm will be presented. Appreciation of AKI as an ICU syndrome and creation of an appropriately matching and sophisticated diagnostic platform of injury assessment are possible and represent the next step in AKI management.

## Introduction

Acute kidney injury (AKI) continues to be an epidemic in patients admitted to the intensive care unit (ICU) (1–4). Across age range (from neonates to adults), regardless of illness severity, and independent of socioeconomic factors, critically ill patients suffering AKI have increased hospital resource utilization (mechanical ventilation, length of stay), higher costs of care, and increased rate of death (1, 5). Significant academic effort has been placed into improved earlier recognition and prediction of disease, either through the tradition markers of serum creatinine (SCr) and/or urine output (UOP) or more novel biomarkers in the urine or serum. Despite nearly two decades of data, however, very few new assessment techniques have gained acceptance and integration into practice (6). Part of the reason may be a near monocular focus on biomarker prediction of AKI progression or AKI-related mortality—two outcomes which are confounded by myriad other factors. AKI does not occur in isolation. The drivers of AKI and the end-organ effects of AKI, like other ICU syndromes such as sepsis and “ARDS,” extend beyond the kidney itself, are multidimensional and change over time (7). Like sepsis and ARDS, management is supportive and aimed at preventing further injury if possible. Finally, there remain no consistently effective, definite therapy for AKI—as there are no true restorative or curative therapies for sepsis and ARDS. In sepsis and ARDS, a significant focus of attention is now placed on refining the phenotype of injury and identifying characteristic manifestations of the syndrome(s) amenable to intervention and trackable over time (8). This is possible for AKI as well. In this minireview, a contemporary approach to biomarker characterization will be discussed. A dynamic and multidimensional approach to AKI, using an AKI biomarker composite (ABC) panel over time, will be presented as a versatile theoretic construct usable to characterize and phenotype AKI itself, refining the precision of diagnosis and making possible the ability to track different aspects of the injury as they change over time. The dynamic assessment would facilitate a focus on the *process* of management, similar to how sepsis and ARDS are assessed and managed. Shifting the focus in this way would potentially increase the opportunities for AKI biomarkers to demonstrate importance in clinical management. Given the increasing broader recognition of both AKI and associated complications, a contemporary and renewed approach to the injury syndrome is warranted.

## One Point And One Outcome: The Limitations Of Static Assessment

Over two decades of research in biomarker research has failed to result in a consensus opinion on the value of incorporating novel diagnostics into routine practice (6, 9). Meta-analyses of biomarker data yield information with limited individual-specific clinical applicability (9, 10). A majority of the studies included in such analyses investigate a single biomarker measured at a single timepoint using the metric of predictive discrimination [area under curve–receiver operating characteristics (AUC-ROC)] to evaluate predictive performance for AKI progression, use of renal replacement therapy, and/or mortality. The AUC-ROC data available, however, identify very few biomarkers with consistently excellent performance (AUC-ROC > 0.85–0.90) for prediction of the three separate outcomes or any individual outcome across multiple populations. Problematically, the comparisons between biomarkers are used to identify the “best” biomarker, with the implication that the best marker would be not only broadly applicable but also the parallel of troponin for acute coronary syndrome—sensitive to injury, responsive to degree of damage, and specific for type of injury (11). There are strengths and weaknesses with this approach. Numerous models of experimental or clinical AKI have identified a number of putative biomarkers, both in the urine and serum (12). Using a consistent outcome(s) leads to a generalizable understanding of the performance of a biomarker vs. other biomarkers (i.e., frame of reference). In addition, picking consistent outcome(s) allows adjudication of the performance of that biomarker across different populations of interest. Unfortunately, there are significant limitations to the current approach. The biomarkers themselves have been mapped to reflect different locations of injury or mechanisms of injury within the kidney, but the predicted outcomes do not reflect this etiologic or “geographic” heterogeneity (13, 14). The individual biomarkers demonstrate marked variation in kinetic profile in relation to injury—rate of rise, magnitude of elevation in relation to purported injury, and rate of decay of detectable biomarker concentration (7, 15, 16). However, using a single point in time does not consider how these biomarkers change over time. Available data would suggest, however, that the change in biomarker concentration can be correlated with phase of AKI (onset, progression, resolution). Together, biomarkers have not been commonly used to subtype or phenotype AKI (thereby refining the precision of diagnosis) but to predict AKI diagnosed by changes in SCr or UOP. Meanwhile, consensus expert opinion has explicitly delineated the importance of improving the precision of AKI diagnosis and, conversely, moving beyond the imprecision of using SCr or UOP alone for delineating functional vs. tubular damage associated AKI (17). In addition, comparison of biomarker AUC-ROC values between studies often does not typically involve statistical tests for superiority (i.e., which test is “better”). Finally, the conclusion of many individual studies and meta-analyses highlighting the biomarker(s) demonstrating the highest predictive performance stops short of offering suggestions of how management itself can change. The implementation is for diagnosis or prognosis only, rarely to guide therapy, or even predict response to therapy (theragnosis). Amidst the numerous meta-analyses, summary statements, and reviews on AKI biomarkers, over 200 biomarkers have been studied in some capacity in human populations—ranging across age and illness. The proportion of these data are notable—for the predominance of a small subset of the discovered markers (~ 10/200) and focus on certain populations of interest (Supplementary Figure 1).

The lack of proven therapeutic options and reliance on supportive management, an inherently reactive strategy, is partially a result of the limited diagnostic tools used in practice. Although stratification systems such as RIFLE, AKIN, and KDIGO have made possible the identification of AKI epidemiology and outcomes, there remains considerable skepticism about what AKI actually is Devarajan (18). Meaning, what injury is actually being predicted by the stratification system-based scores, by changes in creatinine concentration? Just as sepsis and ARDS syndromes are complex and unlikely to be completely described (either predicted or characterized) by a single marker such as fever, white blood cell count, or oxygen saturation, it would be illogical to presume that the complex biology of AKI could be comprehensively ascertained by a single biomarker. Yet, the diagnosis and monitoring of AKI, has largely been dependent on two determinants [change in serum creatinine (SCr) from baseline and then from day to day or tiered amounts of urine output] (Supplementary Table 1). The importance of urine output assessment has only recently been highlighted (19, 20). These markers carry known limitations—including only affording the ability to identify AKI broadly and without precision to type of injury (12).

Taken together, the existing diagnostic paradigm is imprecise, poorly applicable to a complex and changing disease process akin to a syndrome, and not capable of helping guide management. Novel diagnostics have, unfortunately, been tested in the exact system as existing diagnostics, which has hindered identification of their potential for prognosis, diagnosis, and theragnosis.

## Precision In Aki: Combinations And Sequential Biomarkers

The management of critically ill patients is improved by diagnostics specific for the type of injury and the ability to rely on these tests to mirror the recovery or progression of injury. Problematically, the concordance of SCr change with renal injury, the mainstay of alerting a provider to the presence or development of kidney insult, is fraught with limitation, particularly in younger and smaller patients. Reliance on a substandard diagnostic has propagated misclassification of AKI into broad, outdated, and imprecise umbrella categories of “pre-renal” or “intrinsic” AKI. For example, “pre-renal” theoretically defines, in one term, severity, timing, duration, and reversibility of injury while simultaneously underscoring the recommended therapy (i.e., fluid administration). A patient with congestive heart failure, however, would be diagnosed as having pre-renal AKI but violates the aforementioned descriptions of injury and could be significantly harmed by such a one-size-fits-all approach. Furthermore, the pathobiology of pre-renal AKI, often attributed to volume depletion or ischemic AKI, demonstrates marked heterogeneity in acute gene dysregulation and adaptive or maladaptive protein expression in the kidney (18). There is also limited histological evidence supporting the dogma equating intrinsic AKI to acute tubular necrosis (21).

The diagnosis of AKI is being refined. The Acute Dialysis and Quality Initiative (ADQI) international consensus panels have been instrumental in shifting the current paradigm. The 10th ADQI advocated improving the precision of AKI nomenclature using more pathophysiological terms such functional or tubular damage-associated AKI (13) and using a combination of biomarkers to refine the biology related to damage. The combination of a functional marker (SCr) with a tubular damage biomarker such as urinary neutrophil gelatinase associated lipocalin (uNGAL) have been validated in several populations to separate functional vs. damage-associated AKI (22–24). In addition, the classification of “sub-clinical” AKI, damage without measurable changes in SCr, has been made possible and is associated with worse overall outcomes, both with regards to kidney function and overall patient status (22, 25–27). The 16th ADQI recommended study (including risk scores, functional markers, and use of biomarkers) to identify, predict, and further characterize patients with persistent AKI (i.e., ≥48 h of SCr elevation or oliguria) and to consider SCr elevation with return to baseline in the first 48 h to be considered separately from actual AKI (28). Risk stratification systems such as the renal angina index can identify the patients with the highest pretest probability (risk) for evolution into severe AKI after 72 h (29). Meanwhile, matching aberrancies in hemodynamic and bioenergetic drivers of AKI with the kidney's response to insult, as assessed by the adaptive or maladaptive responses to injury, has opened the door to diagnostics matching the phase of illness (30).

Biomarkers demonstrate time-dependent profiles reflective of injury pathology. A broad adult study of multiple biomarkers following cardiac surgery demonstrated unique temporal profiles of the most commonly cited individual biomarkers: NGAL, interleukin-18 (IL-18), kidney injury molecule-1 (KIM-1), and liver-fatty acid binding protein (31–33). An important conclusion of these data and more recent individual population study data is the concordance of specific biomarkers for specific characteristics of AKI. For instance, the profile of KIM-1 appears to be reflective of AKI with high risk of chronic kidney disease (34, 35); in fact, follow-up studies of patients with cardiac surgery associated AKI demonstrate persistent elevation of KIM-1 in patients with chronic kidney disease following AKI. The temporal profile of a biomarker reflective of kidney “stress,” the cell-cycle arrest, marker tissue inhibitor of matrix metalloproteinase-2/insulin-like growth factor binding protein-7 demonstrates marked variation in relation to different drivers of potential AKI (i.e., nephrotoxins, non-steroidal anti-inflammatory agents, and cardiac surgery) (36, 37). Finally, case examples of sequential uNGAL identifies fluid-based AKI phenotypes reflective of not only AKI diagnosis and prognosis but also theragnosis (38). The negative or positive deflection of the biomarker appears to be predictive, specifically, of response to diuretic therapy and function of the tubule for clearance of solute and fluid.

In the absence of novel biomarkers, simply assessing changes in SCr or UOP in a new manner may yield informative, actionable information. SCr in the mathematical construct of kinetic estimated glomerular filtration rate offers insight as to trajectory of filtration injury or recovery (39, 40). Adjustment of the kinetic estimated glomerular filtration rate for total body volume, often significantly labile in critically ill patients, may further refine the prognostic value of this methodology (41). Correction of SCr for fluid balance and as the fluid balance changes may delineate the independent effects of AKI and fluid overload (FO), identifying unique AKI-FO phenotypes (42, 43). Recent data from both adults and children demonstrate the importance of close monitoring of urine output early in ICU course (19, 20). As accumulation of fluid can be a proxy for reduced UOP, and evidence indicates excessive positive fluid balance (FO) is associated with poor outcome (44), attention to how UOP and FO change over time may offer a point of intervention earlier than changes in SCr. To this end, the furosemide stress test (FST), a standardized metric to gauge urine flow after a single diuretic dose, may be valuable to phenotype renal reserve and tubular function (36, 45, 46).

In total, diagnostic assessment of AKI should mirror the pathology of the syndrome. The biology of AKI is manifest in different segments of the nephron and via different mechanistic underpinnings. The manifestation of AKI itself varies considerably as well. Currently, the focus rests squarely upon clearance of solute and fluid, but a significant body of evidence supports the extrarenal distant organ effects of isolated AKI—demonstrating wide ranging physiological perturbation. Following biomarkers in a multiplicative fashion and as they change over time will likely facilitate a deeper understanding of what injury is actually occurring under the umbrella diagnosis of AKI and potentially the trajectory of these injuries.

## A Dynamic Multidimensional Approach To AKI: The AKI Biomarker Composite

ICU management is multidimensional and dynamic. The care for a patient suffering sepsis or respiratory failure is guided by not only patient exam and context but also a series of diagnostic evaluations that occur in multiplicative fashion over the time of ICU course. As an example, respiratory failure from ARDS is assessed, managed, and treated in a sequential, iterative way. Multiple diagnostic inputs ranging from physical exam, radiography, blood gas assessment, capnography, and pulse oximetry are used over time to personalize the approach to a patient. Unique interventions are also specifically directed toward improving oxygenation, augmenting ventilation, reducing secretion load, and mitigating bronchospasm—all within the paradigm of monitoring the syndrome as it changes over time using a diagnostic platform that concurrently changes. Similarly, to track the progression of septic shock, markers such as lactate and central venous oxygen saturation (S_v_O_2_) are followed longitudinally; measurement at a single timepoint only does not allow for adjudication of management or make it possible to track effects of therapy (Table 1). A dynamic diagnostic approach to AKI, mirroring the approaches used for respiratory failure or septic shock, may ultimately lead to more precise and effective therapeutic options.

**Table 1:** Comparison of common diagnostics used for ICU syndromes.

**Syndrome**	**Prevalence in ICU patients**	**Effective management**	**Risk stratification**	**Diagnosis**	**Surveillance and** **therapeutic monitoring**
Sepsis	5–10%	Antibiotics Early Goal Directed Therapy	APACHE-III SOFA, qSOFA PRISM I, II, III, IV PELOD 1, 2 PIM 1, 2	Mental Status Temperature HR, RR MAP WBC Lactate SvO2 Blood Culture/GS CSF Cx/GS Urine Cx/Analysis	Physical Exam Lactate pH Base deficit or excess S_v_O_2_ Urine Output Coagulation Profile Echocardiogram C-reactive protein ESR Procalcitonin NIRS Oximetry Cytokine profile IVC POCUS
ARDS	6–10%	Low tidal volume ventilation Neuromuscular blockade Prone Positioning	Berlin Criteria OI S/F ratio P/F ratio Co-morbidity	CXR Chest CT S_p_O_2_ P_a_O_2_ Echocardiogram Sputum Culture	CXR S_p_O_2_ pCO_2_ pH Lung Ultrasound Respiratory Secretions
AKI	25–30%		Creatinine	Creatinine Urine Output	Creatinine Urine Output

*Common ICU injury syndromes with associated characteristics related to prevalence, management and detection. APACHE-III = Acute Physiology, Age, Chronic Health Evaluation. SOFA, Sequential Organ Failure Assessment; PRISM, Pediatric Risk of Mortality; PELOD, Pediatric Logistic Organ Dysfunction; PIM, Pediatric Index of Mortality; HR, heart rate; RR, respiratory rate; MAP, mean arterial pressure; WBC, white blood cell count; SvO_2_, venous oxygen saturation; GS, gram stain; CSF, cerebrospinal fluid; Cx, culture; ESR, erythrocyte sedimentation rate; NIRS, near infrared spectroscopy; IVC POCUS, inferior vena cava point of care ultrasound; OI, oxygenation index; S/F, saturation of oxygen/fraction of inspired oxygen; P/F, partial pressure of oxygen/fraction of inspired oxygen; CXR, chest radiograph; CT, computed tomograph; SpO_2_, oxygen saturation; pCO_2_, partial pressure of carbon dioxide*.

**Figure 1:**
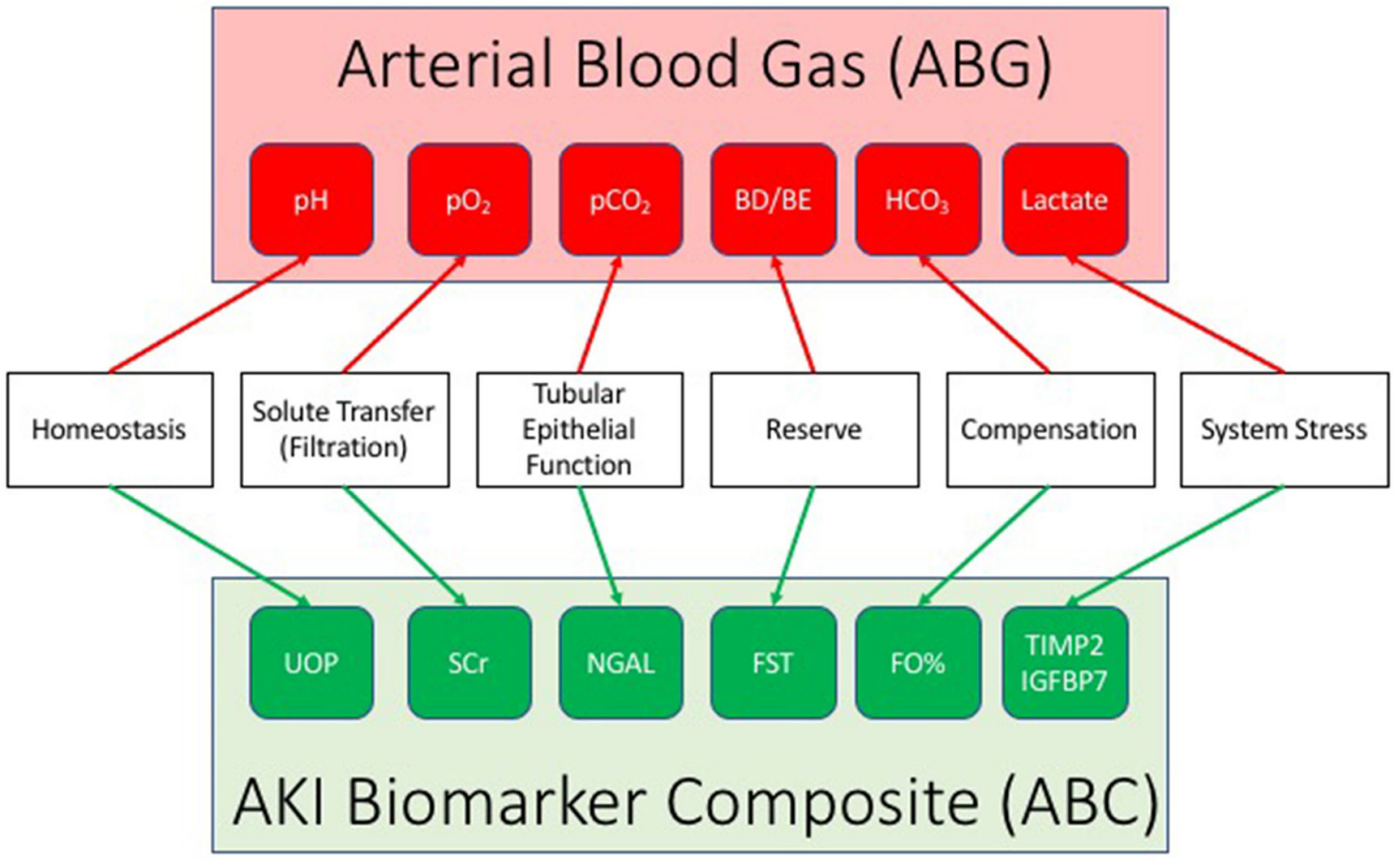
The AKI biomarker composite (ABC). Drawing a parallel to the arterial blood gas, the ABC integrates a series of individual AKI biomarkers to facilitate the status and progression of acute kidney injury (AKI). An AKI biomarker composite integrates a marker of homeostasis (urine output: UOP), filtration (serum creatinine: SCr), tubular function (urine neutrophil gelatinase associated lipocalin: NGAL), renal reserve (furosemide stress test: FST, renal functional reserve: RFR), renal compensation (percent fluid overload: FO%), and stress (tissue inhibitor of matrix metalloproteinase-2/insulin like growth factor binding protein 7: TIMP2/IGFBP7).

An AKI biomarker panel may facilitate simultaneous patient monitoring and targeting. Akin to a blood gas assessment for the purposes of tracking and managing respiratory failure, an ABC can be constructed to parallel biology and mechanistic characteristics of the injury (Figure 1). Urine output reflects homeostasis and overall organ function, while SCr serves as a reflection of filtration. This parallels a blood gas: pH is used as the first arbiter of homeostasis in respiratory failure, while partial pressure of oxygen (pO_2_) is a marker of filtration of oxygen along the alveolar–capillary endothelial border. A marker of tubular epithelial injury mirrors the marker in the lungs of alveolar epithelial function—the ability to exchange gas (O_2_ for carbon dioxide—CO_2_). Damage markers such as uNGAL may identify renal tubular epithelial cell dysfunction, similar to the imputation of alveolar epithelial functionality determined by an arterial partial pressure of carbon dioxide (pCO_2_). The furosemide stress test as mentioned earlier reflects renal functional reserve and can be utilized as a functional capacitance marker, identifying the amount of reserve left in the renal system. Incorporation of FO% into the composite is a real-time assessment of compensation in relation to AKI, potentially an analog to the base excess or deficit on a blood gas. Finally, tissue inhibitor of matrix metalloproteinase-2^*^insulin-like growth factor binding protein-7 may identify varying levels of renal stress, just as serum lactate is used for shock to adjudicate the balance between supply and demand in the setting of oxygen metabolism. The ABC offers the possibility of identifying how much stress exists on the system, the change from homeostatic conditions, aberrancies within the system for clearance of fluid and solute, and how much renal reserve exists—and does so concurrently—as opposed to testing individual biomarkers in isolation and/or at a single point in time. Although untested at this time, and theoretic in nature, this multidimensional combination of markers used simultaneously and over time may provide a dynamic system for tracking AKI—prognosis, diagnosis, and theragnosis. The concept could be implemented in a manner analogous to the use of arterial blood gas sampling for the purposes of respiratory failure—iterative to guide intervention on the ventilator (e.g., fluid balance management) or as an adjudication of a daily trend. Significant work will be required to demonstrate validity to this approach; however, the justification remains simple—the existing diagnostic paradigm is simply too generic, imprecise, unsophisticated, and cannot reasonably be expected to match the heterogeneity and complexity of AKI.

The AKI syndrome affects critically ill patients of all ages and requires a personalized medicine approach. A contemporary and appropriately personalized diagnostic paradigm is possible, practical, and may ultimately identify opportunities to target and manage specific aspects of injury in real time.

## Conclusion

In summary, critically ill patients suffering from AKI need a modern and personalized approach to care. Use of a conventional and one-size-fits-all diagnostic approach to AKI will likely perpetuate the poor outcomes associated with AKI. The potential exists to refine the understanding of AKI and improve diagnostics using sophistication and precision. A dynamic and multimodal approach to AKI, paralleling the approach used for other critical illnesses, may make it possible to identify newer and targeted therapeutic possibilities in the future.

## Author Contributions

The author confirms being the sole contributor of this work and has approved it for publication.

### Conflict of Interest

The author declares that the research was conducted in the absence of any commercial or financial relationships that could be construed as a potential conflict of interest. The handling editor declared DA a past co-authorship with the author RB.
