# Supplementary material for: Dynamic Biomarker Assessment: A Diagnostic Paradigm to Match the AKI Syndrome
Source: Front Pediatr. 2020 Jan 21;7:535. doi: 10.3389/fped.2019.00535 (PMC6986245; doi:10.3389/fped.2019.00535)
Supplement: Supplementary file 1 [file Table_1.DOCX]

Supplemental Table 1. Diagnostic Criteria for Acute Kidney Injury

| Era | Marker |
| --- | --- |
| 1960s | Creatinine |
| 1970s | Creatinine |
| 1980s | Creatinine |
| 1990s | Creatinine |
| 2000s | Creatinine, Urine output |
| 2010s | Creatinine, Urine output |

| Type of AKI | Marker |
| --- | --- |
| Pre-Renal | Creatinine, Urine output |
| Intrinsic | Creatinine, Urine output |
| Post-Renal | Creatinine |

| Location of Injury | Marker |
| --- | --- |
| Glomerular | Creatinine, Urine output |
| Proximal Tubule | Creatinine, Urine output |
| Loop of Henle | Creatinine, Urine output |
| Distal Convoluted Tubule | Creatinine, Urine output |
| Collecting Duct | Creatinine, Urine output |
| Interstitium | Creatinine, Urine output |

| Etiology of Injury | Marker |
| --- | --- |
| Hypoperfusion, Ischemia-Reperfusion | Creatinine, Urine output |
| Oxidative Stress | Creatinine, Urine output |
| Necrosis | Creatinine, Urine output |
| Mitochondrial Failure/Bioenergetic | Creatinine, Urine output |
| Inflammatory | Creatinine, Urine output |
