# Supplementary figures and images for: Dynamic Biomarker Assessment: A Diagnostic Paradigm to Match the AKI Syndrome
Source: Front Pediatr. 2020 Jan 21;7:535. doi: 10.3389/fped.2019.00535 (PMC6986245; doi:10.3389/fped.2019.00535)

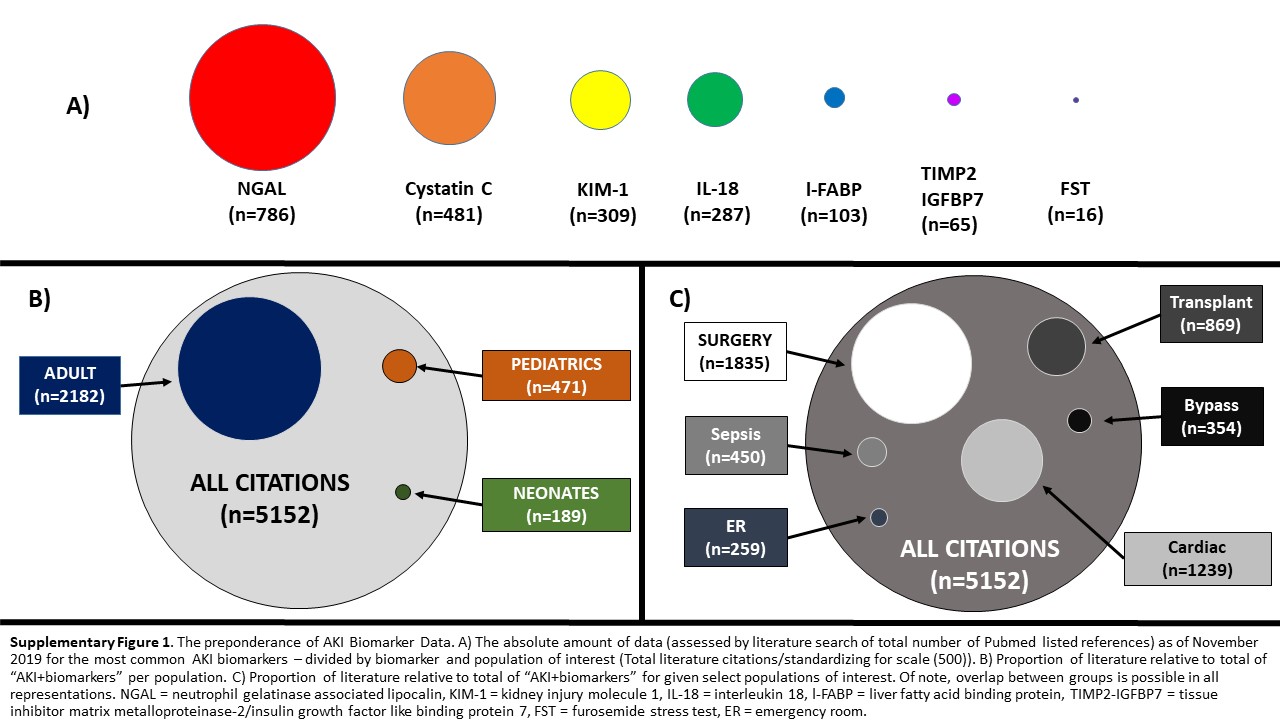

Supplement: Supplementary file 2 [file Image_1.JPEG]
